# Cone -beam computed tomographic (CBCT) analysis of root canal volume changes with OneShape, standard Mani -silk and BlueFlex acer file: An *in vivo * study

**DOI:** 10.6026/973206300222895

**Published:** 2026-05-31

**Authors:** Mandeep Kaur, Renu Bala Sroa, Baljeet Kumar

**Affiliations:** 1 Department of Conservative Dentistry & Endodontics, Government Dental College & Hospital, Amritsar, Punjab, India

**Keywords:** Root canal preparation, canal volume change, OneShape file, standard Mani-silk file, BlueFlex acer file, cone-beam computed tomography (CBCT), 3D reconstruction, nickel-titanium files

## Abstract

Over-instrumentation can result in excessive and inappropriate dentin removal, especially in root canals with complex internal anatomy. Therefore, it is of interest to evaluate changes in root canal volume of permanent mandibular premolars after preparation with OneShape, Standard Mani-Silk and Blueflex Acer file using CBCT and 3D reconstruction. Hence, 45 teeth were divided into three groups, instrumented with the respective file systems and pre- and post-instrumentation canal volumes were measured using Blue Sky Plan and MeshMixer software. The results showed that all file systems significantly increased canal volume (p<0.001), with Blueflex Acer file producing the highest volume change, followed by OneShape and Standard Mani-Silk the least. Standard Mani-Silk showed the best preservation of canal anatomy, suggesting its suitability for conservative endodontic preparation.

## Background:

Modern endodontic therapy is guided by the classical endodontic triad which aims to achieve complete debridement, sterilization of the root canal system and three-dimensional obturation for long-term treatment success [1]. Optimal endodontic preparation should preserve the original morphology of root canals, but the complex internal anatomy-characterized by variable cross-sectional shapes and curvatures-poses significant challenges in cleaning, disinfection and shaping [2]. Deviation from the natural curvature during instrumentation can result in excessive and inappropriate dentin removal, which adversely affects treatment outcomes [3]. The introduction of nickel-titanium (NiTi) rotary instruments has revolutionized endodontic practice by enabling easier, faster and more efficient root canal preparation compared to conventional stainless- steel instruments [4]. Design improvements, including varying tapers, non-cutting safety tips and modifications in blade geometry, have led to new generations of instruments with superior shaping ability and enhanced safety [5]. In addition, thermo mechanical treatments of NiTi alloys, along with variations in kinematics such as reciprocating motion and single-file systems, have further simplified instrumentation while reducing the risk of instrument fracture and cross-contamination [6]. The OneShape file system (MicroMega, Besançon, France) is a single rotary file manufactured from austenite 55-NiTi alloy, operating in continuous rotation. Its electro polished surface, high flexibility and variable cross-sectional geometry provide efficient cutting, superior apical progression and preservation of canal shape [7]. However, studies have shown that it tends to remove more dentin from the coronal third compared to reciprocating systems such as RECIPROC and WaveOne [8]. The Mani-Silk file system (Mani Prime Dental Products, Japan) is a two-file system with a teardrop cross-section and positive rake angle, designed to reduce the "screwing-in" effect. Its heat-treated NiTi alloy improves flexibility, cutting efficiency and fracture resistance. When used in reciprocation, Mani-Silk files demonstrate better centering ability and less canal transportation compared to some rotary systems [9]. Nonetheless, certain studies report greater canal transportation and reduced centering ability in the apical third compared to ProTaper and V-Taper files [10]. A newer system, the Blueflex Acer file, utilizes blue wire technology and is approved by the U.S. FDA. It demonstrates high resistance to cyclic fatigue, excellent cutting efficiency and unmatched flexibility [11]. The file features a non-cutting safety tip to reduce apical perforation and a variable pitch design to prevent ledging, locking and apical extrusion. Manufacturers claim that it minimizes dentin damage and significantly reduces the risk of instrument breakage [12]. Several methodologies have been employed to evaluate the shaping ability of endodontic instruments, including radiography, histological sectioning, stereomicroscopy, scanning electron microscopy, computed tomography (CT) and micro-CT [13]. Among these, cone-beam computed tomography (CBCT) offers a non- invasive, three-dimensional imaging approach with advantages such as high-resolution imaging, lower radiation dose, shorter scan time and cost-effectiveness [14]. CBCT enables accurate measurement of canal volume, surface area and morphological alterations before and after instrumentation. Although numerous studies have analyzed canal transportation and centering ability of different file systems, very few *in vivo* studies have specifically assessed three-dimensional canal volume changes using CBCT [15]. Therefore, it is of interest to evaluate the in vivo changes in canal volume after root canal preparation with three different file systems-One Shape, Mani-Silk and Blueflex Acer file-using CBCT and 3D reconstruction.

## Methodology:

**Figure 1:**
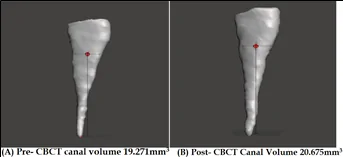
3-dimensional root canal reconstruction and change in the root canal volume before and after instrumentation using software blueskyplan (GROUP I)

**Figure 2:**
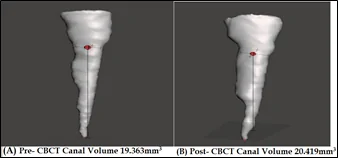
3-dimensional root canal reconstruction and change in the root canal volume before and after instrumentation using software Blueskyplan (GROUP II)

**Figure 3:**
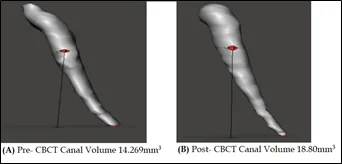
3-dimensional root canal reconstruction and change in the root canal volume before and after instrumentation using software Blueskyplan (GROUP III)

This prospective *in vivo* experimental study was conducted in the Department of Conservative Dentistry and Endodontics, Punjab Government Dental College and Hospital, Amritsar, after obtaining ethical clearance from the Dental Research Ethics Committee affiliated to Baba Farid University of Health Sciences, Faridkot (Approval No. BFUHS/2k22/p-TH/766). A total of 45 systemically healthy patients requiring root canal treatment in permanent mandibular premolars were selected based on inclusion criteria: single-rooted teeth with one straight canal, canal curvature <25°C, mature apices, patients aged 18-45 years and teeth with no previous restorations or endodontic treatment. Exclusion criteria included grossly carious teeth, fractured teeth, abnormal anatomy, incomplete root formation, resorption, calcified canals, periodontal pockets >4 mm, or systemic disorders. Informed consent was obtained from all patients. Pre-instrumentation CBCT scans were taken using PaX-i3D Green™ PHT-60CFO (Vatech Co. Ltd., Seoul, South Korea) with parameters of 95 KVP, 6 mA, 0.08 x 0.08 x 0.08 mm resolution, 50 x 50 mm field of view and ∼10 sec scan time. Images were stored in DICOM format and measurements were standardized at 3 mm (apical third), 5 mm (middle third) and 7 mm (coronal third) from the apex. Following prophylaxis with chlorhexidine rinse and administration of inferior alveolar nerve block (2% lignocaine with 1:200,000 adrenalines); the teeth were isolated with a rubber dam. Standard endodontic access was prepared using a round bur and Endo-Z bur and canal patency was checked with a No.10 K-file. Working length was determined using Root ZX apex locator (J Morita, Japan) and confirmed radiographically. The teeth were randomly allocated into three groups (n=15 each). Group I: canals were prepared using OneShape (MicroMega, France) file (size 25/0.06) in continuous rotation at 400 rpm and 2.0 Ncm torque. Group II: canals were prepared with Standard Mani-Silk files (Mani, Japan) (sizes 25/0.08, 20/0.06, 25/0.06) in reciprocating motion at 500 rpm and 3.0 Ncm torque. Group III: canals were prepared with Blueflex Acer file (Bluedent, India) (sizes 17/0.04, 20/0.04, 20/0.06, 25/0.04, 25/0.06) at 300 rpm and 2.0 Ncm torque. In all groups, instrumentation was performed with 3 mm amplitude pecking motion, with cleaning of the file after every three strokes. Irrigation protocol included 3 ml of 5.25% sodium hypochlorite after each cycle, followed by a final rinse with 10 ml of distilled water. Post-instrumentation CBCT scans were obtained with the same parameters as baseline. Pre- and post-instrumentation DICOM images were imported into BlueSky Plan software (BlueSkyBio, USA) for pulp segmentation and reconstruction in axial, coronal and sagittal planes. Segmented STL models were exported to MeshMixer software (Autodesk, USA) for canal volume analysis from the cementoenamel junction to the apex. The canal volumes were calculated in mm^3^ and tabulated (Figure 1, Figure 2, Figure 3).

## Results:

The present study evaluated the changes in root canal volume before and after preparation with three different file systems-OneShape, Standard Mani-Silk and Blueflex Acer file using CBCT and 3D reconstruction. Intra-group comparison using paired t-test revealed a statistically highly significant increase in canal volume following instrumentation in all three groups (p<0.001). Group I (OneShape) showed a mean canal volume increase of 3.534 mm^3^, while Group II (Standard Mani-Silk) exhibited a lower mean increase of 2.714 mm^2^. Group III (Blueflex Acer file) demonstrated the maximum canal enlargement; with a mean volume change of 6.659 mm^3^ (Table 1). Inter-group comparison of percentage canal volume increase was performed using One-Way ANOVA. Results showed a statistically significant difference among the groups (p=0.001). The highest percentage increase in canal volume was observed in Group III (Blueflex Acer file) (28.799% ± 13.4%), followed by Group I (OneShape) (17.870% ± 7.3%) and Group II (Standard Mani-Silk) (14.440% ± 9.4%) (Table 2). Overall, the results indicate that all three file systems were effective in increasing root canal volume; however, Blueflex Acer file achieved the most substantial enlargement and demonstrated superior shaping ability when compared to OneShape and Standard Mani-Silk (Figure 1, Figure 2, Figure 3). The data were statistically analyzed using paired Student's t-test for intra-group comparison, one-way ANOVA for inter-group comparison and Tukey's HSD post-hoc test for pairwise comparison. A p value <0.05 was considered statistically significant.

## Discussion:

The primary goal of chemo-mechanical root canal preparation is to clean and shape the canal effectively while preserving the natural anatomy of the tooth. Selection of a file system must consider canal curvature, morphology, dentin thickness and preservation of the coronal structure. The advancement of nickel-titanium (NiTi) rotary and reciprocating instruments has improved efficiency, but their shaping ability and influence on canal volume remain important factors in clinical practice [10]. In the present study, CBCT and 3D reconstruction were employed to evaluate canal volume changes after preparation with three file systems-One Shape, Standard Mani-Silk and Blueflex Acer file. The findings demonstrated that all three systems produced a significant increase in canal volume after preparation, confirming their efficiency in shaping. However, there was a clear difference in the extent of dentin removal among the groups. Blueflex Acer file showed the highest mean canal volume change (6.659 mm^3^), followed by OneShape (3.534 mm^3^) and Standard Mani-Silk (2.714 mm^3^). The percentage volume change was also greatest with Blueflex Acer file (28.799%), compared with OneShape (17.870%) and Mani-Silk (14.440%). The results of the present study are consistent with earlier reports Elashiry*et al.* [11] observed a significant increase in canal volume with the OneShape file, highlighting its efficiency in shaping. Similarly, Yuan and Yang [12] reported substantial canal enlargement with OneShape, although their study found less canal transportation compared to other rotary systems. In contrast, Tambe *et al*. [13] demonstrated that WaveOne reciprocation maintained centering ability better than OneShape, indicating that shaping outcomes may vary depending on anatomical complexity and instrumentation technique. Mani-Silk files, which use a tear-drop cross section and reciprocating motion, produced the least change in canal volume in the present study. This is in agreement who reported that Mani-Silk tends to produce less dentin removal but may cause minor canal transportation in the apical region [14]. Deepthi *et al.* [15] also found Mani-Silk to exhibit superior centering ability compared to other systems, supporting the present study's observation that Mani-Silk preserved canal morphology with minimal enlargement. Blueflex Acer file demonstrated the greatest increase in canal volume. This could be attributed to its advanced heat-treated blue wire metallurgy, triangular cross-section and variable pitch design, which together enhance flexibility, cyclic fatigue resistance and cutting efficiency. The present findings align with those of Bucheli *et al.* [16], who reported that file systems with advanced metallurgy and continuous rotation (such as RECIPROC Blue) achieved greater canal enlargement than those with conventional designs. Statistical analysis in this study showed that all three groups produced highly significant differences in canal volume before and after preparation (p<0.001). Intergroup analysis revealed significant differences between Blueflex Acer file and both OneShape and Mani-Silk, while the difference between OneShape and Mani-Silk was not significant. This suggests that Blueflex Acer file provides more aggressive shaping compared to the other two systems. Similar intergroup differences were also observed in the Tukey post-hoc test. From a clinical perspective, excessive dentin removal may compromise root strength and predispose teeth to fracture [17]. While Blueflex Acer file demonstrated the largest canal enlargement, its aggressive shaping may not always be advantageous, particularly in roots with thin dentinal walls. In contrast, Mani-Silk, by preserving more dentin, may offer greater structural integrity and safety in clinical situations [18]. OneShape represents an intermediate approach, balancing efficiency and conservation of tooth structure [19, 20]. The present study has some limitations. It was conducted on a relatively small sample size and limited to mandibular premolars with mild curvature (<25°C). Results may vary in molars or more curved canals. Additionally, long- term clinical outcomes such as fracture resistance, sealing ability and success of obturation were not evaluated. Future studies incorporating a larger sample size, diverse tooth types and long-term follow-up are needed. In conclusion, the present in vivo study demonstrates that Blueflex Acer file provides the greatest increase in canal volume, followed by OneShape and Standard Mani-Silk. However, considering the importance of preserving dentin and maintaining canal anatomy, Mani-Silk may be recommended in cases where conservation is a priority. File selection should therefore be guided not only by shaping efficiency but also by anatomical considerations and long-term tooth survival.

## Conclusion:

All three file systems-OneShape, Standard Mani-Silk and Blueflex Acer file-effectively increased root canal volume after preparation. Blueflex Acer file showed the highest canal volume increase, followed by OneShape, while Standard Mani-Silk preserved canal anatomy with minimal volume change. Thus, Standard Mani-Silk is preferred when conservation of root canal structure is desired.

## Figures and Tables

**Table 1:** Comparison of canal volume before and after preparation using paired‘t' Test

**Group**	**Mean Canal Volume Before (mm^3^)**	**Mean Canal Volume After (mm^3^)**	**Mean Difference (mm^3^)**	**p-value**
I (One Shape File)	16.318 ± 2.539	19.852 ± 2.274	-3.534	0.001 (HS)
II (Standard Mani Silk File)	15.289 ± 1.941	18.003 ± 2.377	-2.714	0.003 (HS)
III (Blueflex Acer file)	14.314 ± 3.142	20.973 ± 6.598	-6.659	0.001 (HS)
HS= Highly Significant (p <0.001)

**Table 2:** Percentage canal volume increase using one-way ANOVA

**Group**	**N**	**Mean % Volume Increase**	**SD**	**95% CI**	**Minimum**	**Maximum**
I (One Shape File)	15	17.87	7.303	13.826 - 21.915	6.791	31.377
II (Standard Mani Silk File)	15	14.44	9.408	9.230 - 19.650	3.191	31.392
III (Blueflex Acer file)	15	28.799	13.41	21.372 - 36.228	9.596	68.289
Significance level:
P < 0.05 (significant);
<0.001 (highly significant);
>0.05 (non-significant)
